# *Drosophila melanogaster* c306 GAL4 is not specific to ovarian cells

**DOI:** 10.17912/micropub.biology.001383

**Published:** 2024-11-11

**Authors:** Ashley C. Goll, Kaden H. Bex, Tina L. Tootle

**Affiliations:** 1 Biology, University of Iowa, Iowa City, Iowa, United States

## Abstract

A widely used genetic tool in *Drosophila melanogaster* is the GAL4/UAS system. This bipartite system allows for tissue and cell-specific expression and is used for both overexpression and RNA interference (RNAi) knockdown studies. Here we provide evidence that c306 GAL4 expression is not restricted to somatic ovarian cells but is also expressed in cells giving rise to adult bristles. Future work is needed to fully define the cell-specific expression pattern c306 GAL4.

**Figure 1. c306 GAL4 driven RNAi knockdown of Fascin:**
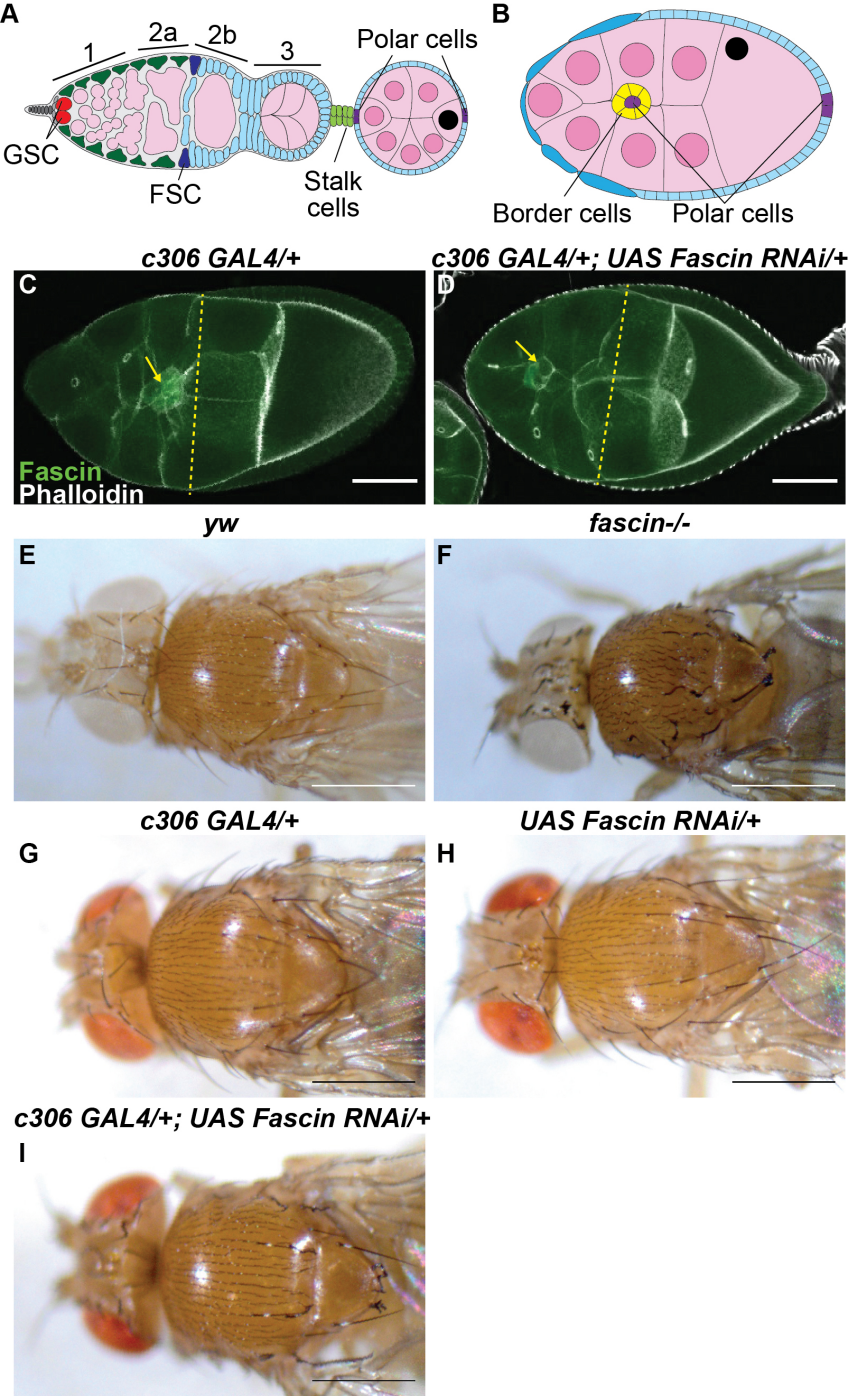
A. Schematic of germarium and budded follicle: Germline stem cell (GSC) niche cells (gray), GSCs (red), germline cysts (pink, region 1-3), escort cells (green), follicle stem cells (FSCs; dark blue), follicle cells (blue), stalk cells (light green), polar cells (purple), nurse cells in budded follicle (pink with pink nuclei), oocyte in budded follicle (pink with black nucleus). B. Schematic of Stage 9 follicle: polar cells (purple), border cells (yellow), follicle cells (blue), nurse cells (pink with pink nuclei), oocyte (pink with black nucleus). C-D. Maximum projects of two confocal slices of Stage 9 follicles of the indicated genotype stained for F-actin (phalloidin, white) and Fascin (green); *c306 GAL4/+* (C) and *c306 GAL4/+; Fascin RNAi/+* (D). Yellow arrows indicate border cell cluster and yellow dashed line indicates on-time migration. Scale bars = 50μm. E-I. Dissecting scope images of adult female fly heads and thoraxes of the indicated genotypes; scale bars = 0.2mm. *yw* (E), *fascin ^sn28^ /fascin ^sn28^* (F), *c306 GAL4/+* (G), *UAS Fascin RNAi/+* (H) and *c306 GAL4/+; UAS Fascin RNAi/+* (I). *Fascin RNAi* = HMS02450. Prior studies report that c306 GAL4 is expressed in the FSCs, follicle cells in region 3 of the germarium, stalk cells, polar cells, and border cells (A-B). Driving RNAi knockdown of Fascin with c306 GAL4 decreases Fascin levels within the border cells and results in delayed migration (C-D). Loss of Fascin results in a “singed” bristle phenotype in adults (E-F). c306 GAL4 driven RNAi knockdown of Fascin results in a subset of the bristles appearing “singed” (G-I).

## Description

In *Drosophila* , the GAL4/UAS system is widely used to control tissue and cell-type specific expression (Brand & Perrimon, 1993) . GAL4 is a transcription factor and binds to the UAS sequence, driving the expression of whatever is placed downstream. Thus, the system can be used to increase gene expression when the coding sequence is downstream of the UAS or knockdown when the downstream sequences drive RNAi (Duffy, 2002; Matsushima et al., 2007; Ni et al., 2008) . The cell/tissue specificity of the system is controlled by the enhancer regulating the expression of GAL4. Many of the early GAL4 lines were generated by random integration of a P-element containing the GAL4 sequence, allowing the GAL4 expression to be controlled by the enhancer it inserted near (Brand & Perrimon, 1993) . Defining the tissue and cell-type specificity of each GAL4 requires expressing a UAS-reporter construct and assessing expression in different tissues and developmental times (Deng et al., 1997; Goentoro et al., 2006; Manseau et al., 1997) . Thus, many GAL4 line expression patterns are not fully characterized.

Here we describe our unexpected finding that c306 GAL4 expression is not restricted to specific ovarian somatic cells. The c306 GAL4 driver was generated by a random integration screen (Manseau et al., 1997) . Initial and subsequent characterization of this line using UAS-reporter constructs revealed it is expressed in a subset of the somatic cells during *Drosophila* oogenesis (Chang et al., 2013; Furriols et al., 2007; Manseau et al., 1997) . Each *Drosophila* ovary is comprised of ~15-20 ovarioles or chains of sequentially developing follicles or egg chambers (Giedt & Tootle, 2023) . Follicle development begins in the germarium, which houses both germline stem cells and somatic, follicle stem cells, which give rise to all the cells of the follicle (Fig 1A). In region 3 of the germarium, 16-cell germline cysts (15 nurse cells and one oocyte) are surrounded by follicle cells. A subset of the follicle cells differentiates into polar cells, which specify the anterior and posterior of each follicle, and stalk cells, which separate the developing follicles from one another (Fig 1A). These follicles progress through 14 stages of development. In Stage 9, additional subsets of follicle cells are specified, including the border cells (Fig 1B). The two anterior polar cells recruit 6-8 follicle cells to become border cells. The border cell cluster (including the two polar cells), then collectively migrates between the nurse cells toward the oocyte and ultimately contributes to the formation of the micropyle (Montell, 2003; Montell et al., 1992; Montell et al., 2012) . The c306 GAL4 driver is expressed in the follicle stem cells and early undifferentiated follicle cells in the germarium prior to being restricted to the stalk and polar cells in the budded follicles or egg chambers. During Stage 9, c306 GAL4 is also expressed in the border cells (Chang et al., 2013; Furriols et al., 2007; Manseau et al., 1997) .

During our studies on the actin bundling protein Fascin, *Drosophila* Singed, in border cell migration (Lamb et al., 2020; Lamb et al., 2021) , we found that the c306 GAL4 is also expressed in cells that will give rise to adult bristles. Specifically, when c306 GAL4 is used to knockdown Fascin using RNAi, we not only see knockdown in the border cells which results in delayed migration (Fig 1C-D) but find that it impacts bristle formation. Fascin is called Singed in *Drosophila* because loss of Fascin results in “singed” looking bristles (Fig 1E-F) (Cant et al., 1994) . To our surprise, when c306 GAL4 is used to knockdown Fascin, we find that some bristles also exhibit a “singed” phenotype ( Fig. 1I ); the GAL4 and RNAi only controls exhibit normal bristles (Fig 1G-H). These data indicate that the c306 GAL4 driver is expressed in the cells generating the bristles. Future work is needed to define the cell-specific expression of c306 GAL4 during bristle development.

## Methods

***Drosophila* genetics**

Fly stocks were maintained on cornmeal/agar/yeast food at 22°C except where noted. The following stocks were obtained from the Bloomington Drosophila Stock Center: *yw* (BDSC 1495), *c306 GAL4* (BDSC 3743) and *fascin* *RNAi* HMS02450 (BDSC 42615). The *fascin ^sn28^* line was provided by Jennifer Zanet (Université de Toulouse, Toulouse, France; (Zanet et al., 2012) ). Expression of the RNAi line for follicle analyses was achieved by crossing to *c306 GAL4* , maintaining the fly crosses at 22°C and maintaining progeny for border cell analyses at 29°C for 3 days. Before immunofluorescence staining, newly eclosed flies were fed wet yeast paste every day for 3 days.

**Immunostaining**

*Drosophila* ovaries (5-8 pairs per sample) were dissected in room temperature Grace’s insect medium (Lonza). Ovaries were fixed for 10 min with 4% paraformaldehyde diluted in Grace’s medium. Samples were washed six times for 10 min each at room temperature in antibody wash (1X phosphate-buffered saline [PBS], 0.1% Triton X and 0.1% bovine serum albumin). The primary antibody, mouse anti-Fascin 1:50 (sn7c, Cooley, L., AB_528239; (Cant et al., 1994) ), was obtained from the Developmental Studies Hybridoma Bank (DSHB) which was developed under the auspices of the National Institute of Child Health and Human Development and maintained by the Department of Biology, University of Iowa (Iowa City, IA). The primary antibody was diluted in antibody wash and incubated overnight at 4°C. After six washes in antibody wash (10 min each), samples were incubated overnight at 4°C in secondary antibody AF488::goat anti-mouse (AB_2534069; ThermoFisher Scientific) diluted 1:500 in antibody wash. Alexa Fluor 647-conjugated phalloidin (1:250; A22287; ThermoFisher Scientific: Invitrogen) was included in both primary and secondary antibody incubations. Following six washes in antibody wash (10 min each), samples were then rinsed in 1X PBS and mounted on slides in 1 mg/mL phenylenediamine in 50% glycerol, pH 9 (Platt & Michael, 1983) .

**Imaging**

Microscope images of immunostained *Drosophila* follicles were taken using LAS SPE Core software on a Leica TCS SPE mounted on a Leica DM2500 using ACS APO 20x/0.6 IMM Corr -/D objective (Leica Microsystems). Microscope images of adult *Drosophila* females maintained at 22°C were obtained with an Olympus DP25 camera mounted on an SZ61 using Olympus cellSens software. Maximum projections, merge, rotation, and cropping were performed using ImageJ software (Abramoff et al., 2004) .

**Figure generation**

Illustrator (Adobe,RRID: SCR 010279) was used to generated the schematics and assemble the figure.

## Reagents

**Table:** 

**Organisms/Strains**			
***Drosophila* Strains **	**Genotype**	**Identifier**	**Available from**
*yw*	y ^1^ w ^1^	RRID: BDSC_1495	Bloomington Drosophila Stock Center (BDSC)
*c306 GAL4*	P{w[+mW.hs]=GawB}c306, w[1118]	RRID: BDSC_3743	BDSC
*UAS Fascin RNAi*	y[1] sc[*] v[1] sev[21]; P{y[+t7.7] v[+t1.8]=TRiP.HMS02450} attP2	RRID: BDSC_42615	BDSC
* fascin ^sn28^ / fascin ^sn28^*	sn28/sn28	FBal0046496	from J. Zanet

**Antibodies**			
**Name**		**Identifier**	**Source**
Monoclonal mouse anti-Singed sn7C		RRID: AB_528239	Developmental Studies Hybridoma Bank
Goat anti-Mouse IgG (H+L) Cross-Absorbed Secondary Antibody, Alexa Fluor ^TM^ 488		RRID: AB_2534069	ThermoFisher Scientific

**Reagents**			
**Name**		**Identifier**	**Source**
647 Phalloidin		Cat# A22287	ThermoFisher Scientific: Invitrogen
bovine serum albumin		Cat# A7284	Sigma-Aldrich
Grace's Insect Media		Cat# 04-457F	Lonza

## References

[R1] Abramoff MD, Magalhaes PJ, Ram SJ. 2004. Image processing with ImageJ. Biophotonics Int. 11: 36-42. 20.

[R2] Brand AH, Perrimon N (1993). Targeted gene expression as a means of altering cell fates and generating dominant phenotypes.. Development.

[R3] Cant K, Knowles BA, Mooseker MS, Cooley L (1994). Drosophila singed, a fascin homolog, is required for actin bundle formation during oogenesis and bristle extension.. J Cell Biol.

[R4] Chang YC, Jang AC, Lin CH, Montell DJ (2013). Castor is required for Hedgehog-dependent cell-fate specification and follicle stem cell maintenance in Drosophila oogenesis.. Proc Natl Acad Sci U S A.

[R5] Deng WM, Zhao D, Rothwell K, Bownes M (1997). Analysis of P[gal4] insertion lines of Drosophila melanogaster as a route to identifying genes important in the follicle cells during oogenesis.. Mol Hum Reprod.

[R6] Duffy JB (2002). GAL4 system in Drosophila: a fly geneticist's Swiss army knife.. Genesis.

[R7] Furriols M, Ventura G, Casanova J (2007). Two distinct but convergent groups of cells trigger Torso receptor tyrosine kinase activation by independently expressing torso-like.. Proc Natl Acad Sci U S A.

[R8] Giedt MS, Tootle TL (2023). The Vast Utility of Drosophila Oogenesis.. Methods Mol Biol.

[R9] Goentoro LA, Yakoby N, Goodhouse J, Schüpbach T, Shvartsman SY (2006). Quantitative analysis of the GAL4/UAS system in Drosophila oogenesis.. Genesis.

[R10] Lamb MC, Anliker KK, Tootle TL (2020). Fascin regulates protrusions and delamination to mediate invasive, collective cell migration in vivo.. Dev Dyn.

[R11] Lamb MC, Kaluarachchi CP, Lansakara TI, Mellentine SQ, Lan Y, Tivanski AV, Tootle TL (2021). Fascin limits Myosin activity within Drosophila border cells to control substrate stiffness and promote migration.. Elife.

[R12] Manseau L, Baradaran A, Brower D, Budhu A, Elefant F, Phan H, Philp AV, Yang M, Glover D, Kaiser K, Palter K, Selleck S (1997). GAL4 enhancer traps expressed in the embryo, larval brain, imaginal discs, and ovary of Drosophila.. Dev Dyn.

[R13] Matsushima Y, Adán C, Garesse R, Kaguni LS (2007). Functional analysis by inducible RNA interference in Drosophila melanogaster.. Methods Mol Biol.

[R14] Montell DJ (2003). Border-cell migration: the race is on.. Nat Rev Mol Cell Biol.

[R15] Montell DJ, Rorth P, Spradling AC (1992). slow border cells, a locus required for a developmentally regulated cell migration during oogenesis, encodes Drosophila C/EBP.. Cell.

[R16] Montell DJ, Yoon WH, Starz-Gaiano M (2012). Group choreography: mechanisms orchestrating the collective movement of border cells.. Nat Rev Mol Cell Biol.

[R17] Ni JQ, Markstein M, Binari R, Pfeiffer B, Liu LP, Villalta C, Booker M, Perkins L, Perrimon N (2007). Vector and parameters for targeted transgenic RNA interference in Drosophila melanogaster.. Nat Methods.

[R18] Platt JL, Michael AF (1983). Retardation of fading and enhancement of intensity of immunofluorescence by p-phenylenediamine.. J Histochem Cytochem.

[R19] Zanet J, Jayo A, Plaza S, Millard T, Parsons M, Stramer B (2012). Fascin promotes filopodia formation independent of its role in actin bundling.. J Cell Biol.

